# SARS-CoV-2 intra-host diversity, antibody response, and disease severity after reinfection by the variant of concern Gamma in Brazil

**DOI:** 10.1038/s41598-023-33443-1

**Published:** 2023-05-05

**Authors:** Felipe Gomes Naveca, Valdinete Alves Nascimento, Fernanda Nascimento, Maria Ogrzewalska, Alex Pauvolid-Corrêa, Mia Ferreira Araújo, Ighor Arantes, Érika Rocha Batista, Alessandro Álvares Magalhães, Fernando Vinhal, Tirza Peixoto Mattos, Irina Riediger, Maria do Carmo Debur, Beatriz Grinsztejn, Valdiléa G. Veloso, Patrícia Brasil, Rodrigo Ribeiro Rodrigues, Darcita Buerger Rovaris, Sandra Bianchini Fernandes, Cristiano Fernandes, João Hugo Abdalla Santos, Lígia Fernandes Abdalla, Rubens Costa-Filho, Marineide Silva, Victor Souza, Ágatha Araújo Costa, Matilde Mejía, Maria Júlia Brandão, Luciana Fé Gonçalves, George Allan Silva, Michele Silva de Jesus, Karina Pessoa, André de Lima Guerra Corado, Debora Camila Gomes Duarte, Ana Beatriz Machado, Ketiuce de Azevedo Zukeram, Natalia Valente, Renata Serrano Lopes, Elisa Cavalcante Pereira, Luciana Reis Appolinario, Alice Sampaio Rocha, Luis Fernando Lopez Tort, Tsuyoshi Sekizuka, Kentaro Itokawa, Masanori Hashino, Makoto Kuroda, Filipe Zimmer Dezordi, Gabriel Luz Wallau, Edson Delatorre, Tiago Gräf, Marilda Mendonça Siqueira, Gonzalo Bello, Paola Cristina Resende

**Affiliations:** 1 Laboratório de Ecologia de Doenças Transmissíveis na Amazônia, Instituto Leônidas e Maria Deane, Fiocruz, Manaus, Brazil; 2 Laboratório de Flavivírus, Instituto Oswaldo Cruz, Fiocruz, Rio de Janeiro, Rio de Janeiro Brazil; 3 Laboratory of Respiratory Viruses and Measles, Oswaldo Cruz Institute (IOC), Oswaldo Cruz Foundation (FIOCRUZ), Rio de Janeiro, RJ Brazil; 4 Department of Veterinary Integrative Biosciences, Texas A&M University, College Station, TX USA; 5 Laboratório de AIDS e Imunologia Molecular, Instituto Oswaldo Cruz, FIOCRUZ, Rio de Janeiro, Brazil; 6 Secretaria de Saúde de Aparecida de Goiânia, Goiás, Brazil; 7 Laboratório HLAGYN, Goiânia, Goiás Brazil; 8 Laboratório Central de Saúde Pública do Amazonas (LACEN-AM, Manaus, Amazonas Brazil; 9 Laboratório Central de Saúde Pública do Paraná (LACEN-PR) Curitiba, Paraná, Brazil; 10 Instituto Nacional de Infectologia Evandro Chagas (INI), Fiocruz, Rio de Janeiro, Brazil; 11 Laboratório Central de Saúde Pública do Espírito Santo (LACEN-ES), Vitória, Espirito Santo Brazil; 12 Laboratório Central de Saúde Pública do Estado de Santa Catarina (LACEN-SC), Florianópolis, Santa Catarina Brazil; 13 Fundação de Vigilância em Saúde do Amazonas-Dra Rosemary Costa Pinto, Manaus, Amazonas Brazil; 14 Hospital Adventista de Manaus, Manaus, Amazonas Brazil; 15 Universidade do Estado do Amazonas, Manaus, Brazil; 16 Hospital Pró-Cardíaco-Rede Américas UHG, Rio de Janeiro, Brazil; 17 CENUR Litoral Norte, Universidad de la República, Salto, Uruguay; 18 Pathogen Genomics Center, National Institute of Infectious Diseases, 1-23-1 Toyama, Shinjuku-Ku, Tokyo, 162-8640 Japan; 19 Instituto Aggeu Magalhães, Fundação Oswaldo Cruz, Recife, Pernambuco Brazil; 20 Departamento de Biologia, Centro de Ciências Exatas, Naturais e da Saúde, Universidade Federal do Espírito Santo, Alegre, Brazil; 21 Instituto Gonçalo Moniz, Fundação Oswaldo Cruz, Salvador, Brazil

**Keywords:** SARS-CoV-2, Molecular biology

## Abstract

The rapid spread of the SARS-CoV-2 Variant of Concern (VOC) Gamma in Amazonas during early 2021 fueled a second large COVID-19 epidemic wave and raised concern about the potential role of reinfections. Very few cases of reinfection associated with the VOC Gamma have been reported to date, and their potential impact on clinical, immunological, and virological parameters remains largely unexplored. Here we describe 25 cases of SARS-CoV-2 reinfection in Brazil. SARS-CoV-2 genomic analysis confirmed that individuals were primo-infected with distinct viral lineages between March and December 2020 (B.1.1, B.1.1.28, B.1.1.33, B.1.195, and P.2) and reinfected with the VOC Gamma between 3 to 12 months after primo-infection. We found a similar mean cycle threshold (Ct) value and limited intra-host viral diversity in both primo-infection and reinfection samples. Sera of 14 patients tested 10–75 days after reinfection displayed detectable neutralizing antibodies (NAb) titers against SARS-CoV-2 variants that circulated before (B.1.*), during (Gamma), and after (Delta and Omicron) the second epidemic wave in Brazil. All individuals had milder or no symptoms after reinfection, and none required hospitalization. These findings demonstrate that individuals reinfected with the VOC Gamma may display relatively high RNA viral loads at the upper respiratory tract after reinfection, thus contributing to onward viral transmissions. Despite this, our study points to a low overall risk of severe Gamma reinfections, supporting that the abrupt increase in hospital admissions and deaths observed in Amazonas and other Brazilian states during the Gamma wave was mostly driven by primary infections. Our findings also indicate that most individuals analyzed developed a high anti-SARS-CoV-2 NAb response after reinfection that may provide some protection against reinfection or disease by different SARS-CoV-2 variants.

## Introduction

The COVID-19 epidemic trajectory has been influenced by the immune landscape generated by SARS-CoV-2 infections^1,2^. Two COVID-19 epidemic waves severely hit the Brazilian state of Amazonas during the first year of the pandemic, the first one associated with the dissemination of multiple SARS-CoV-2 B.1.* lineages and the second one driven by the SARS-CoV-2 Variant of Concern (VOC) Gamma^3,4^. Consequently, Amazonas displayed the highest absolute decline in life expectancy at birth from 2019 to 2020 among all Brazilian states^2^. Some studies estimated that a high proportion of the population in Amazonas (> 70%) had already been infected with SARS-CoV-2 by October 2020 and speculated a high proportion of reinfection among new cases during the Gamma wave^5,6^. Other studies, by contrast, estimated a much lower SARS-CoV-2 seroprevalence in Amazonas in late 2020 (< 40%)^7–11^, suggesting that reinfections by Gamma did not play a significant role in driving the second COVID-19 epidemic wave.

A deeper understanding of the clinical and virological characteristics of reinfections with the VOC Gamma may provide essential clues about the potential role of reinfection on the past, current, and future trajectory of the SARS-CoV-2 epidemic in Amazonas and Brazil. However, only three cases of reinfection with the VOC Gamma were documented in Brazil^12–14^. Furthermore, it is also important to better understand the potential impact of SARS-CoV-2 reinfection on cross-neutralizing immunity response and intra-host viral diversity. Evidence from breakthrough infections suggests that multiple antigen exposures improve the potency and breadth of serum neutralizing antibodies (NAb) against SARS-CoV-2 variants^15–19^ and may also increase the complexity of intra-host viral mutant spectra^20^. This phenomenon raises the possibility that replication in the face of a pre-existing anti-SARS-CoV-2 immunity during reinfections may improve the NAb response against different SARS-CoV-2 variants while selecting for new mutations with the potential to become dominant at the epidemiological level.

In this study, we described the clinical and virological characteristics of 25 SARS-CoV-2 reinfections with the VOC Gamma in subjects from six different Brazilian states who had been primo-infected between 3 to 12 months earlier. The complexity of intra-host SARS-CoV-2 quasispecies was assessed at both the first and second COVID-19 episodes. We also collected serum samples after reinfection and tested for plaque reduction neutralization (PRNT) against viral variants that circulate before (B.1.1.28 and B.1.1.33), during (Gamma), and after (Delta and Omicron) the second epidemic wave in Brazil.

## Methods

### Reinfection cases and ethical aspects

In this study, we include 25 cases of adults living in four different regions of Brazil, including West-Central (n = 13), South (n = 7), North (n = 3), and Southeast (n = 2), that presented two episodes of COVID-19 with at least 90 days apart. The first and second episodes occurred between March and December 2020, and December 2020 and June 2021, respectively. All patients had nasopharyngeal and oropharyngeal swabs (NPS) collected in viral transport media (VTM) and tested by SARS-CoV-2 real-time Reverse Transcriptase—Polymerase Chain Reaction (RT-PCR) in their respective State Health Departments as part of the official network of the Brazilian Ministry of Health for the diagnostic and surveillance of SARS-CoV-2. Samples of patients who were SARS-COV-2 positive by real-time RT-PCR twice within at least 90 days apart were sent to the National Reference Laboratory for reinfection investigation and confirmation, according to the Technical Note 52/2020-CGPNI/DEIDT/SVS/MS^21^. This study was approved by the Ethics Committee of the Amazonas State University (CAAE: 25430719.6.0000.5016) and by the Ethics Committee of FIOCRUZ (CAAE: 68118417.6.0000.5248), which waived signed informed consent for all participants. All methods followed guidelines and regulations of the Brazilian Ministry of Health.

### SARS-CoV-2 real-time RT-PCR confirmation and genomic sequencing

Suspected reinfection samples were sent to one of the sequencing hubs of the COVID-19 Fiocruz Genomic Surveillance Network (http://www.genomahcov.fiocruz.br, LVRS, Fiocruz, Rio de Janeiro; ILMD, Fiocruz Amazonas; or HLAGyn, Goiás) to have the total nucleic acid extracted from the VTM specimens by Maxwell^®^ RSC Viral Total Nucleic Acid Purification Kit (Promega, Madison, WI) or QIAmp RNA viral mini kit (Qiagen). Then, immediately submitted to a real-time RT-PCR designed to amplify nucleocapsid phosphoprotein (N) gene of SARS-CoV-2^22^ or EDx kit Biomanguinhos protocol to amplify the envelope protein (E) gene^23^. Using the nucleic acid extracts, we generated the whole genome of SARS-CoV-2 by the in-house amplicon sequencing protocols described^24,25^ but with some improvements in the primer scheme (Supplementary File) or the Illumina COVIDSeq test kit (Illumina) with some adaptations^26^. Libraries were produced with Nextera XT or COVIDSeq and sequenced with MiSeq Reagent Micro Kit v2 (300-cycles). The FASTQ reads were obtained following the Illumina pipeline on BaseSpace, imported into Geneious v10.2.6, trimmed (BBDuk 37.25), or into CLC Genomic Workbench (Qiagen), and mapped (BBMap 37.25) against the reference sequence EPI_ISL_402124 available in EpiCoV database from GISAID (https://www.gisaid.org/). Consensus sequences with a mean read depth of 1341 × were generated after excluding duplicate reads.

### Genomic analyses

PANGO lineages were assigned to all sequences by the Pangolin algorithm^27^, and later confirmed using maximum likelihood (ML) phylogenetic analyses. SARS-CoV-2 complete genome sequences from all cases were aligned with high quality (< 1% of unidentified bases “N”) SARS-CoV-2 whole-genomes (> 29 kb) of representative lineages retrieved from the EpiCoV database at GISAID. The final dataset of each variant to which primo-infection and reinfection sequences were previously assigned was obtained by clusterization of their GISAID complete datasets using CD-HIT v.4.8.1^28^.The resulting dataset was aligned by MAFFT v7.467^29^ and subjected to a ML phylogenetic analysis with IQ-TREE v2.1.2^30^ under the best nucleotide substitution model selected by the ModelFinder application^31^. The branch support was assessed by the approximate likelihood-ratio test based on the Shimodaira–Hasegawa-like procedure (SH-aLRT) with 1000 replicates^32^. The genomic analysis of intra-host single nucleotide variants (iSNV) was performed with ViralFlow v.0.0.6^33^ as described in detail elsewhere^34^ for 22 of the 25 reinfection cases. Only genomes with more than 95% coverage breadth and 100 reads of average coverage depth were considered for minor variant analyses. Five nucleotides of reads boundaries were clipped, regions with Phred score lower than 20 were removed and reads smaller than 75 nucleotides were excluded from the analysis. Only mutations that appear in both sense and antisense reads with a frequency above 5% of total reads at each position and a depth of at least 100 reads were defined as iSNV^34^.

#### Serological analyses

Fourteen serum samples collected 10–75 days after the second episode of COVID-19 were tested for SARS-CoV-2-specific neutralizing antibodies (NAb) by plaque reduction neutralization test (PRNT_90_). We also include serum samples collected 3–21 days after hospitalization from 30 individuals primo-infected with the Gamma variant. For PRNT_90_, an aliquot of serum sample inactivated at 56 °C for 30 min was tested in VERO CCL-81 cells in duplicate at serial two-fold dilutions to determine 90% endpoint titers against four infectious SARS-CoV-2 lineages, including the reference strains B.1.1.33 (EPI_ISL_1181439), B.1.1.28 (EPI_ISL_2645638), Gamma (EPI_ISL_1402431) and Delta (EPI_ISL_2645417) established by the National Reference Laboratory. Serum samples were considered seropositive when a serum dilution of at least 1:10 reduced no less than 90% of the formation of SARS-CoV-2 viral plaques^35^.

### Statistical analyses

The non-parametric Wilcoxon matched-pairs signed-rank test was used to compare multiple samples per subject (real-time RT-PCR Cycle threshold [Ct] of samples from the first and second episodes and level of NAb against different SARS-CoV-2 variants in the plasma taken after reinfection). The Mann–Whitney test was used to compare samples from different groups of individuals. Only Ct values from samples analyzed with the same real-time RT-PCR diagnostic assay were compared. Specimens in which NAb could not be detected (PRNT_90_ < 10) were assigned an arbitrary value of five to include NAb as a continuous variable. The threshold for statistical significance was set to *P* < 0.05. Graphics and statistical analyses were performed using GraphPad v9.02 (Prism Software, United States).

## Results

**Figure 1:**
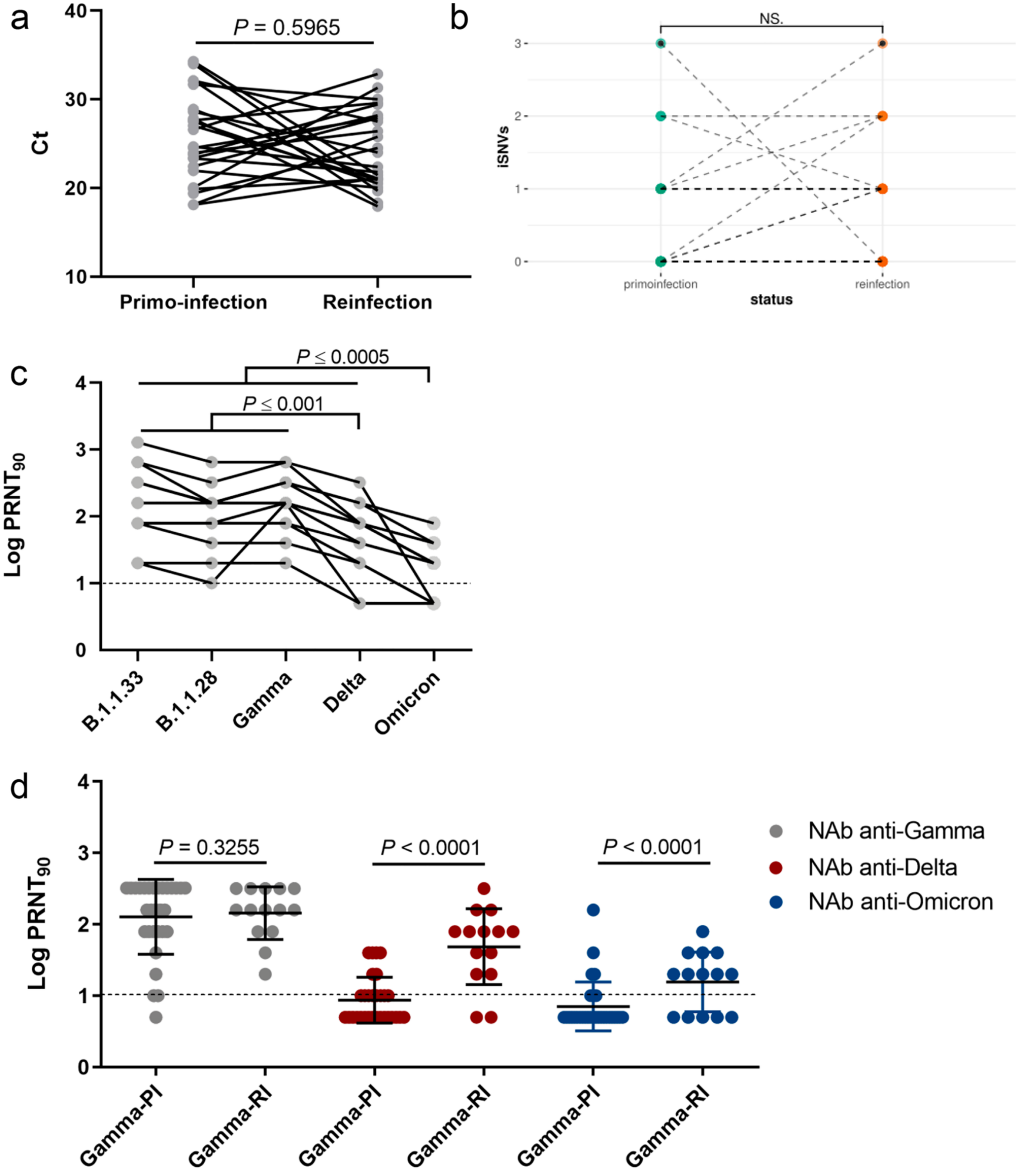
Viral load in NPS samples and neutralization capacity of convalescent serum of reinfected individuals. (**a**) Real-time RT-PCR Ct value distribution of NPS SARS-CoV-2 positive samples taken from 25 individuals at primo-infection and reinfection. Each line represents a single individual. (**b**) Number of iSNVs at primo-infection and reinfection samples. Two-tailed P value was calculated with the Wilcoxon matched-pairs signed-rank test. *NS* Non-significant. (**c**) Individual trajectories of neutralization titers (PRNT_90_) against different SARS-CoV-2 lineages in convalescent plasma from 14 individuals collected 10–75 days following SARS-CoV-2 reinfection. Each line represents a single individual. (**d**) Comparison of neutralization titers (PRNT_90_) against VOCs Gamma, Delta and Omicron in convalescent plasma from hospitalized individuals primo-infected with Gamma (n = 30; Gamma-PI) and individuals reinfected with Gamma (n = 14; Gamma-RI). Horizontal bars represent sample medians and interquartile range. The dashed line indicates the detection limit of the PRNT_90_ assay. Two-tailed P values calculated with the Wilcoxon matched-pairs signed-rank test (**a**,**b**) or the Mann–Whitney test (**c**) are shown.

We analyzed 25 individuals who presented two episodes of COVID-19 within 3 to 12 months (Table 1, Supplementary Fig. S1). They were predominantly female (64%), unvaccinated (92%), and with an age that ranged from 17 to 73 years old. Most cases had no reported comorbidities (80%) and presented mild clinical symptoms (92%), including fever, myalgia, cough, sore throat, nausea, anosmia, ageusia, and back pain in the first episode of COVID-19. Two individuals required hospitalization at primo-infection. Patients had a milder clinical presentation (84%) or were asymptomatic (16%) at the time of sample collection, and none required hospitalization at reinfection. The mean time between symptoms onset and collection date was 4.0 days at the first infection and 3.5 days at the reinfection, and SARS-CoV-2 positive samples displayed real-time RT-PCR Ct values ranging from 18.0 to 34.3 (Table 1). Of note, 14 individuals displayed mean Ct values < 25.0 during the second episode of COVID-19, and nine individuals displayed much lower Ct values in the second than in the first episode (Ct_first_ − Ct_second_ > 3.0). The overall mean Ct value of the first (25.7) and second (24.5) episodes were not significantly different (*P* > 0.05) for the whole group (Fig. 1A).

**Table 1:** Clinical and epidemiological data of Gamma reinfection cases.

Case	Age, gender	Virus name	Acession number GISAID	Depth coverage	Pango lineage	ct	Onset symptoms	Collection date	Clinical outcome 1st COVID-19 episode	Time between first and second infection	Vaccination
1	29,F	hCoV-19/Brazil/AM-FIOCRUZ-20140055FN-R1/2020	EPI_ISL_811148	3283x	B.1.195	27,5	16/03/2020	24/03/2020	Not hospitalized. Recovered	281	No
		hCoV-19/Brazil/AM-FIOCRUZ-20143138FN-R2/2020	EPI_ISL_811149	4663X	P.1	20,5	24/12/2020	30/12/2020	Not hospitalized. Recovered		
2	50,F	hCoV-19/Brazil/AM-FIOCRUZ-20142223MR-R1/2020	EPI_ISL_1114151	1333x	B.1.1	34	16/10/2020	19/10/2020	Not hospitalized. Recovered	92	No
		hCoV-19/Brazil/AM-FIOCRUZ-21140415MR-R2/2021	EPI_ISL_1034304	6452x	P.1	19,7	16/01/2021	19/01/2021	Not hospitalized. Recovered		
3	40,F	hCoV-19/Brazil/AM-FIOCRUZ-20140452 MJ-R1/2020	EPI_ISL_1034305	1063x	B.1.195	19,9	21/04/2020	22/04/2020	Not hospitalized. Recovered	282	No
		hCoV-19/Brazil/AM-FIOCRUZ-21140646 MJ-R2/2021	EPI_ISL_1034306	2077x	P.1	21	asymptomatic	29/01/2021	Not hospitalized. Recovered		
4	24,M	hCoV-19/Brazil/GO-HLAGYN-1031607-R1/2021	EPI_ISL_2017281	1679x	B.1.1.33	24,5	15/08/2020	19/08/2020	Not hospitalized. Recovered	224	No
		hCoV-19/Brazil/GO-HLAGYN-1586463_R2/2021	EPI_ISL_2017282	888X	P.1	27,9	25/03/2021	31/03/2021	Not hospitalized. Recovered		
5	25,F	hCoV-19/Brazil/GO-HLAGYN-1088252-R1/2021	EPI_ISL_2017323	826x	B.1.1	28,6	22/08/2020	01/09/2020	Not hospitalized. Recovered	188	No
		hCoV-19/Brazil/GO-HLAGYN-1520715_R2/2021	EPI_ISL_2017324	764x	P.1	24	01/03/2021	08/03/2021	Not hospitalized. Recovered		
6	36,F	hCoV-19/Brazil/GO-HLAGYN-855822-R1/2020	EPI_ISL_2017449	523x	B.1.1.33	31,7	01/07/2020	05/07/2020	Not hospitalized. Recovered	291	Yes 1st 21/01/2021 2nd 26/02/2021
		hCoV-19/Brazil/GO-HLAGYN-1623988_R2/2021	EPI_ISL_2187985	1193X	P.1	30	20/04/2021	22/04/2021	Not hospitalized. Recovered		
7	39,F	hCoV-19/Brazil/GO-HLAGYN-920573-R1/2020	EPI_ISL_2187989	815X	B.1.1.33	32,1	20/07/2020	22/07/2020	Not hospitalized. Recovered	280	No
		hCoV-19/Brazil/GO-HLAGYN-1633028_R2/2021	EPI_ISL_2187990	925X	P.1	27,5	22/04/2021	28/04/2021	Not hospitalized. Recovered		
8	29,F	hCoV-19/Brazil/GO-HLAGYN-1013302-R1/2020	EPI_ISL_2188000	1080X	B.1.1.33	23,4	09/08/2020	14/08/2020	Not hospitalized. Recovered	262	No
		hCoV-19/Brazil/GO-HLAGYN-1638914_R2/2021	EPI_ISL_2227564	1171x	P.1	28,2	28/04/2021	03/05/2021	Not hospitalized. Recovered		
9	73,M	hCoV-19/Brazil/PR-FIOCRUZ-34071-R1/2020	EPI_ISL_2196362	601x	B.1.1.28	24,67	11/06/2020	18/06/2020	Hospitalized. Recovered	286	No
		hCoV-19/Brazil/PR-FIOCRUZ-21069-R2/2021	EPI_ISL_2196252	348x	P.1	22,38	31/03/2021	31/03/2021	Not hospitalized. Recovered		
10	30,F	hCoV-19/Brazil/PR-FIOCRUZ-33964-R1/2020	EPI_ISL_3061893	1584x	B.1.1.28	18,1	01/10/2020	02/10/2020	Not hospitalized. Recovered	150	No
		hCoV-19/Brazil/PR-FIOCRUZ-16726-R2/2021	EPI_ISL_3061892	883x	P.1.14	21,14	asymptomatic	01/03/2021	Not hospitalized. Recovered		
11	31,M	hCoV-19/Brazil/SC-FIOCRUZ-34264-R1/2020	EPI_ISL_4563059	711x	B.1.1.28	28,88	06/07/2020	10/07/2020	Not hospitalized. Recovered	327	No
		hCoV-19/Brazil/SC-FIOCRUZ-33117-R2/2021	EPI_ISL_4563061	1885x	P.1	21,03	30/05/2021	02/06/2021	Not hospitalized. Recovered		
12	30,M	hCoV-19/Brazil/SC-FIOCRUZ-33864-R1/2020	EPI_ISL_3061901	1217x	B.1.1.28	23,34	09/09/2020	14/09/2020	Not hospitalized. Recovered	162	No
		hCoV-19/Brazil/SC-FIOCRUZ-11195-R2/2021	EPI_ISL_1534003	1708x	P.1	21,81	17/02/2021	23/02/2021	Not hospitalized. Recovered		
13	21,F	hCoV-19/Brazil/SC-FIOCRUZ-34078-R1/2020	EPI_ISL_2196357	1094x	B.1.1.28	21,97	21/09/2020	25/09/2020	Not hospitalized. Recovered	192	No
		hCoV-19/Brazil/SC-FIOCRUZ-22230-R2/2021	EPI_ISL_2196249	3046x	P.1	20,03	01/04/2021	05/04/2021	Not hospitalized. Recovered		
14	37,F	hCoV-19/Brazil/SC-FIOCRUZ-34070-R1/2020	EPI_ISL_2196360	297x	B.1.1.28	18,14	08/08/2020	12/08/2020	Not hospitalized. Recovered	219	No
		hCoV-19/Brazil/SC-FIOCRUZ-20618-R2/2021	EPI_ISL_2196250	163x	P.1	25,79	15/03/2021	19/03/2021	Not hospitalized. Recovered		
15	22,M	hCoV-19/Brazil/SC-FIOCRUZ-34157-R1/2020	EPI_ISL_3061902	639x	B.1.1.33	27,81	21/10/2020	26/10/2020	Not hospitalized. Recovered	150	No
		hCoV-19/Brazil/SC-FIOCRUZ-25336-R2/2021	EPI_ISL_3061900	718x	P.1.2	17,95	22/03/2021	25/03/2021	Not hospitalized. Recovered		
16	51,F	hCoV-19/Brazil/ES-FIOCRUZ-33961-R1/2020	EPI_ISL_3061879	5088x	B.1.1.33	19,38	06/12/2020	09/12/2020	Not hospitalized. Recovered	93	Yes 1st 29/01/21 2nd 09/04/21
		hCoV-19/Brazil/ES-FIOCRUZ-16403-R2/2021	EPI_ISL_2645521	1207x	P.1	24,5	09/03/2021	12/03/2021	Not hospitalized. Recovered		
17	42,M	hCoV-19/Brazil/RJ-FIOCRUZ-1691-R1/2020	EPI_ISL_2196361	2480x	B.1.1.33	32,17	06/04/2020	08/04/2020	Not hospitalized. Recovered	387	No
		hCoV-19/Brazil/RJ-FIOCRUZ-21373-R2/2021	EPI_ISL_2196251	2366x	P.1	18,3	not informed	30/04/2021	Not hospitalized. Recovered		
18	24,F	hCoV-19/Brazil/GO-HLAGYN-826071_R1/2020	EPI_ISL_2497433	882X	B.1.1.33	20,04	22/06/2020	26/06/2020	Not hospitalized. Recovered	320	No
		hCoV-19/Brazil/GO-HLAGYN-1651296_R2/2021	EPI_ISL_2497440	586X	P.1	31,3	asymptomatic	12/05/2021	Not hospitalized. Recovered		
19	32,F	hCoV-19/Brazil/GO-HLAGYN-870588_R1/2020	EPI_ISL_2497434	834X	B.1.1.28	23,75	01/07/2020	09/07/2020	Not hospitalized. Recovered	323	No
		hCoV-19/Brazil/GO-HLAGYN-1680829_R2/2021	EPI_ISL_2497469	900X	P.1	29,43	asymptomatic	28/05/2021	Not hospitalized. Recovered		
20	17,F	hCoV-19/Brazil/GO-HLAGYN-977307_R1/2020	EPI_ISL_2497435	792X	B.1.1.33	26,58	31/07/2020	06/08/2020	Not hospitalized. Recovered	292	No
		hCoV-19/Brazil/GO-HLAGYN-1673804_R2/2021	EPI_ISL_2497459	856X	P.1.7	32,86	20/05/2021	25/05/2021	Not hospitalized. Recovered		
21	47,M	hCoV-19/Brazil/GO-HLAGYN-1216505_R1/2020	EPI_ISL_2617626	1004X	B.1.1.28	23,96	10/10/2020	15/10/2020	Hospitalized. Recovered	223	No
		hCoV-19/Brazil/GO-HLAGYN-1676036_R2/2021	EPI_ISL_2617627	754X	P.1	26,41	22/05/2021	26/05/2021	Not hospitalized. Recovered		
22	55,F	hCoV-19/Brazil/GO-HLAGYN-998600_R1/2020	EPI_ISL_2921603	173x	B.1.1.33	27,04	07/08/2020	11/08/2020	Not hospitalized. Recovered	300	No
		hCoV-19/Brazil/GO-HLAGYN-1700566_R2/2021	EPI_ISL_2921604	573x	P.1	20,92	06/06/2021	07/06/2021	Not hospitalized. Recovered		
23	19,M	hCoV-19/Brazil/GO-HLAGYN-1123866_R1/2020	EPI_ISL_2921605	931x	B.1.1.33	22,44	08/09/2020	11/09/2020	Not hospitalized. Recovered	266	No
		hCoV-19/Brazil/GO-HLAGYN-1696639_R2/2021	EPI_ISL_2921606	935x	P.1	27,89	03/06/2021	04/06/2021	Not hospitalized. Recovered		
24	39,F	hCoV-19/Brazil/GO-HLAGYN-1074287_R1/2020	EPI_ISL_3087898	869X	B.1.1.198	27,65	27/08/2020	28/08/2020	Not hospitalized. Recovered	290	No
		hCoV-19/Brazil/GO-HLAGYN-1715778_R2/2021	EPI_ISL_2921607	837x	P.1.7	29,59	13/06/2021	14/06/2021	Not hospitalized. Recovered		
25	37,M	hCoV-19/Brazil/GO-HLAGYN-1330226_R1/2020	EPI_ISL_2921608	750x	P.2	34,29	15/12/2020	17/12/2020	Not hospitalized. Recovered	182	No
		hCoV-19/Brazil/GO-HLAGYN-1724317_R2/2021	EPI_ISL_2921609	620x	P.1	21,47	14/06/2021	17/06/2021	Not hospitalized. Recovered		

Four patients were asymptomatic at the time of sample collection during reinfection.

Whole SARS-CoV-2 genomes were recovered from all 50 samples analyzed. Most of them were high-quality sequences (< 1% of N) with a few exceptions (< 15% of N) that were recovered from samples with low viral load (Ct > 33). All SARS-CoV-2 genomes recovered contained enough mutations to confidently assign the corresponding SARS-CoV-2 lineage with high support (1.0). The PANGO lineage system indicated the presence of five different SARS-CoV-2 lineages in the first COVID-19 episodes (B.1.1, B.1.1.28, B.1.1.33, B.1.195, B.1.1.198 and P.2). On the other hand, Pangolin assignment identified the unique presence of the VOC Gamma (lineages P.1, P.1.2, P.1.7, P.1.12 and P.1.14) in all second episodes (Table 1), a finding that was confirmed by the ML phylogenetic analysis (Supplementary Fig. S2A,B). This procedure allowed us to conclude that all suspected cases correspond to reinfections with the VOC Gamma. Analysis of the Spike (S) gene of Gamma viruses detected at reinfection reveals the presence of the canonical lineage signatures plus additional mutations L5F and T76I in one P.1 sequence, D178G in one P.1.12 sequence, A522V in one P.1 sequence, and P681H in two P.1.7 sequences (Supplementary Fig. S2C).

To test the potential impact of pre-existing immunity against SARS-CoV-2 on viral evolution within infected hosts, we use a high depth of coverage sequencing to compare iSNV detected during first and second COVID-19 episodes in 22 subjects. Our analysis revealed an overall low number of iSNVs (between 0 and 3) at both primo-infection (mean = 0, stdev = 0.86) and reinfection (mean = 1, stdev = 0.88) and indicates no significant difference (*P* > 0.05) in the average number of point mutations estimated for SARS-CoV-2 quasispecies at first and second COVID-19 episodes (Fig. 1B). Furthermore, most iSNV identified at both primo-infection (58%, 7/12) and reinfection (86%, 6/7) were synonymous and were mostly located outside the Spike coding sequence (CDS), with the only exception of one synonymous iSNV detected in a sample at reinfection. Thus, we found no evidence of increasing SARS-CoV-2 quasispecies diversity or rapid selection of immune escape mutation during reinfection despite viral replication in the face of a pre-existing immunity against SARS-CoV-2 probably mounted after primo-infection.

To analyze the potential impact of reinfection on NAb response, serum samples of 14 patients out of 25 were collected 10–75 days after the second SARS-CoV-2 positive real time RT-PCR and tested for plaque reduction neutralization against ancestral variants (B.1.1.28 and B.1.1.33) and VOCs (Gamma, Delta, and Omicron). These individuals were primo-infected with lineages B.1.1 (*n* = 2), B.1.1.198 (*n* = 1), B.1.1.28 (*n* = 4), B.1.1.33 (*n* = 5), B.1.195 (*n* = 1), and P.2 (*n* = 1). All patients have detectable NAb against lineages B.1.1.28, B.1.1.33 and Gamma, and most patients also have detectable NAb against Delta (86%) and Omicron (64%). The neutralization geometric mean titers against Omicron (PRNT_90_ = 16) and Delta (PRNT_90_ = 49), however, were significantly lower (*P* < 0.05) than for B.1.1.28 (PRNT_90_ = 103), B.1.1.33 (PRNT_90_ = 160) and Gamma (PRNT_90_ = 160) (Fig. 1C). The levels of NAb in reinfected subjects were next compared with a group of 30 hospitalized individuals primo-infected with Gamma. Most hospitalized individuals primo-infected with Gamma displayed NAb against Gamma (97%), but only a minor fraction displayed NAb against Delta (47%) or Omicron (23%). Furthermore, hospitalized subjects primo-infected with Gamma displayed similar levels of NAb against Gamma (PRNT_90_ = 127), but significantly lower levels of NAb against Delta (PRNT_90_ = 9) and Omicron (PRNT_90_ = 7), than reinfected individuals (*P* < 0.0001) (Fig. 1D).

## Discussion

This study describes 25 Brazilian individuals that were primo-infected with SARS-CoV-2 lineages B.1.195 (two cases), B.1.1 (two cases), B.1.1.28 (eight cases), B.1.1.33 (11 cases), B.1.1.198 (one case), and P.2 (one case) between March and December 2020. These subjects were reinfected with the VOC Gamma 3–12 months later (between December 2020 and June 2021). Lineages B.1.1.28 and B.1.1.33 were the most prevalent Brazilian variants between March and October 2020, whereas lineage B.1.1 circulated at low prevalence in Brazil during 2020. The lineage B.1.195 was locally prevalent in the Amazonas state from March to June 2020, and lineage P.2 was highly prevalent in most Brazilian states between November 2020 and February 2021. The VOC Gamma was frequently detected in the Amazonas state since December 2020 and became the most prevalent viral variant across all Brazilian regions from February to July 2021 (http://www.genomahcov.fiocruz.br/dashboard-en/). Thus, the viral lineages here detected at primo-infection and reinfection largely mirror contemporaneous SARS-CoV-2 variants circulating in different Brazilian regions.

The Ct values analyses support comparable viral replication at the upper respiratory tract at both primo-infection and reinfection, agreeing with a previous study^36^. Several studies demonstrated that Ct values, that inversely correlate with the log viral load, are also negatively correlated with cultivable virus^37,38^ and the transmission risk^39,40^. Up to 70% of patients remained positive in cell culture at a Ct ≤ 25^37,38^, and 85% of case-contact pairs with plausible onward transmission had a case Ct < 25^39,40^, suggesting that a Ct ≤ 25 could be used as a reasonable surrogate of infectivity using different in-house RT-PCR methods. Of note, 56% (14/25) of individuals here described displayed a mean Ct value < 25.0 at reinfection with Gamma. These findings support that some individuals display viral loads at reinfection that may have been sufficient to transmit the virus and that reinfections with the VOC Gamma might have contributed to the onward transmission of SARS-CoV-2 in Brazil. These findings, however, do not demonstrate that Gamma reinfections were common and do not provide evidence of wide population-level immune escape for the VOC Gamma in Brazil.

Most patients here analyzed were young (< 50 years old) and displayed mild clinical symptoms in the first COVID-19 episode, characteristics usually associated with low NAb responses^41–43^. We speculate that the mild severity of primary infections may have induced a transient NAb response that substantially decayed by the time of Gamma reinfection^44–47^. A previous study provided estimates of the typical time frame to reinfection for several coronaviruses and predicted that reinfection with SARS-CoV-2 under endemic conditions would likely occur between 3 and 63 months after peak antibody response, with a median of 16 months^48^. All individuals in our study were reinfected between 3 and 12 months after the primo-infections, consistently with the hypothesis of waning humoral immunity. According to this model, reinfection will become increasingly common as the epidemic progresses. This observation, combined with the high transmissibility of the VOC Gamma^3,4^ may explain the more significant number of reinfections reported for Gamma when compared with the few reinfections with non-VOCs previously detected in Brazil^49,50^.

A modeling study predicts that while protection from SARS-CoV-2 infection will wane substantially over a year, protection from severe disease should largely remain^51^. Consistent with this prediction, we observed that all patients had a milder clinical presentation (84%) or were asymptomatic (16%) at the time of sample collection during reinfection, and none required hospitalization. These numbers are consistent with previous studies that demonstrated a shallow risk of severe or lethal SARS-CoV-2 reinfections, much lower than in primary infections, irrespective of the reinfecting viral lineage^52–58^. These findings suggest that natural immunity induced by ancestral SARS-CoV-2 variants should provide high protection against severe illness during Gamma reinfections. Thus, the high number of hospitalizations and deaths and the high case fatality rate (CFR) observed during the Gamma wave in Amazonas could only be explained if primary infections drove most SARS-CoV-2 cases detected. The low CFR observed during the third COVID-19 epidemic wave in Amazonas driven by the Omicron variant^59^, by contrast, entirely agrees with the superinfection hypothesis.

A previous study of breakthrough infections suggests that multiple antigen exposures may increase the complexity of intra-host viral mutant spectra^20^. To test the possibility that replication in the face of a pre-existing anti-SARS-CoV-2 immunity during reinfections may also select for more complex SARS-CoV-2 quasispecies, we analyzed the intra-host genetic diversity in first and second COVID-19 episodes. Our analysis revealed that all samples here analyzed (22 out of 25) harbor a low number of iSNVs (0–3), mostly located outside the Spike. Therefore, there was no significant increase in the complexity of the viral mutant spectra at reinfection compared to that of primo-infection. Our results are in accordance with other studies supporting limited within-host viral diversity (0–6 iSNVs) in acute SARS-CoV-2 infections^34,60–63^ and suggest that most acute reinfections probably did not play a key role in the generation of new viral variants. However, longitudinal analysis of reinfected subjects is critical for a broader understanding of the potential impact of infection-induced immunity on the short-term intra-host SARS-CoV-2 evolution and further studies may be necessary to better address the diversity in SARS-CoV-2 infected subjects.

The impact of reinfection on the potency, breadth, and durability of serum NAb activity against SARS-CoV-2 has not yet been fully established. Sera from 14 patients here collected 10–75 days after reinfection displayed detectable NAb against SARS-CoV-2 lineages that circulated before (B.1.1.28, B.1.1.33), during (Gamma), and after (Delta and Omicron) the second COVID-19 epidemic wave in Brazil. A significant fraction of reinfected patients displayed detectable NAb against the immune escape VOC Omicron (64%), although NAb response against Omicron was much lower than against the other viral variants tested. Interestingly, individuals reinfected with Gamma displayed similar NAb levels against Gamma when compared to individuals with severe primo-infections with Gamma, but higher levels of NAb levels against the VOCs Delta and Omicron. These findings endorse that reinfection boosted the potency and breadth of NAb against different viral variants, similar to that previously observed in individuals with hybrid immunity^16,64,65^. These results also support those reinfected individuals may display more robust protection against reinfection and severe illness by new variants introduced in the population.

Our study has several limitations. First, although Ct values could be used as a surrogate of infectivity, we have no contact-tracing information to demonstrate that reinfected subjects were able to transmit the virus to susceptible individuals. Second, although samples at primo-infection and reinfection were taken at about the same time from onset of symptoms and displayed a similar mean Ct value, we do not have information about temporal intra-host viral replication dynamics during both infection episodes. Third, samples at reinfection were taken within one week from onset of symptoms and significant immune selection pressure may be only detectable at later times. Fourth, our study was also limited by the absence of longitudinal data of anti-SARS-CoV-2 NAb, particularly between the first and the second COVID-19 episodes. Thus, we could not measure the level of NAb immediately before reinfections and confirm if primo-infections effectively induce a transient neutralization response that later wanes over time.

In summary, our findings confirm several cases of reinfection with the VOC Gamma in individuals who had a first symptomatic infection with non-VOCs between 3 to 12 months earlier in Brazil. No significant decrease in viral load, inferred from Ct values, was observed during reinfection compared with primo-infection, suggesting that some Gamma reinfected subjects may have contributed to the onward endemic transmission of SARS-CoV-2. However, the low severity of reinfections here detected, contrasted to the high number of hospitalizations and the high CFR observed during the spread of Gamma in Amazonas, support that reinfections did not drive most infections during the second COVID-19 epidemic wave in that state. Our findings also suggest that SARS-CoV-2 reinfections may boost the breath of NAb and thus increase protection against reinfection with different SARS-CoV-2 variants circulating in the population.

## Supplementary Information

Supplementary Information.

## Data Availability

The consensus SARS-CoV-2 sequences generated in this work are available online at EpiCoV database in GISAID https://www.gisaid.org under the accession numbers: EPI_ISL_811148, EPI_ISL_811149, EPI_ISL_1034304 to 1034306, EPI_ISL_1114151, EPI_ISL_1534003, EPI_ISL_2017281 to 2017324, EPI_ISL_2017449, EPI_ISL_2187985, EPI_ISL_2187989, EPI_ISL_2187990, EPI_ISL_2188000, EPI_ISL_2196249 to 2196252, EPI_ISL_2196357, EPI_ISL_2196360 to 2196362, EPI_ISL_2227564, EPI_ISL_2497433 to 2497435, EPI_ISL_2497440, EPI_ISL_2497459, EPI_ISL_2497469, EPI_ISL_2617626, EPI_ISL_2617627, EPI_ISL_2645521, EPI_ISL_2921603 to 2921609, EPI_ISL_3061879, EPI_ISL_3061892, EPI_ISL_3061893, EPI_ISL_3061900 to 3061902, EPI_ISL_3087898, EPI_ISL_4563059 and EPI_ISL_4563061.
